# Synthesis, crystallographic, spectroscopic studies and biological activity of new cobalt(II) complexes with bioactive mixed sulindac and nitrogen-donor ligands

**DOI:** 10.1186/s13065-017-0268-2

**Published:** 2017-05-10

**Authors:** Asia M. Shalash, Hijazi I. Abu Ali

**Affiliations:** Department of Chemistry, Birzeit University, P.O. Box 14, West Bank, Palestine

**Keywords:** Cobalt(II) complexes, Nitrogen donor ligands, Sulindac, Anti-bacterial activity

## Abstract

Four novel complexes [Co(H_2_O)_4_(sul)_2_] **1**, [Co(2-ampy)_2_(sul)_2_] **2**, [Co(H_2_O)_2_(1,10-phen) (sul)_2_] **3** and [Co(2,9-dimephen)(sul)_2_] **4** (sul = sulindac, 2-ampy = 2-amino pyridine, 1,10-phen = 1,10-phenanthroline and 2,9-dimeph = 2,9-dimethyl-1,10-phenanthroline) were prepared and characterized by IR, UV–Visible spectroscopy and magnetic properties. The crystal structures of complexes **1** and **4** were determined by single-crystal X-ray diffraction. In-vitro anti-bacterial activity for the prepared complexes against Gram-positive (*Staphylococcus epidermidis, Staphylococcus aureus*) and Gram-negative (*Bordetella, Escherichia coli*) bacteria and Yeast species (*Saccharomyces and Candida*) were performed using agar well-diffusion method. Only complex **4** showed reasonable activity against yeast. All compounds showed more anti-bacterial activity against Gram-positive bacteria than Gram-negative.

**Electronic supplementary material:**

The online version of this article (doi:10.1186/s13065-017-0268-2) contains supplementary material, which is available to authorized users.

**Graphical abstract:**
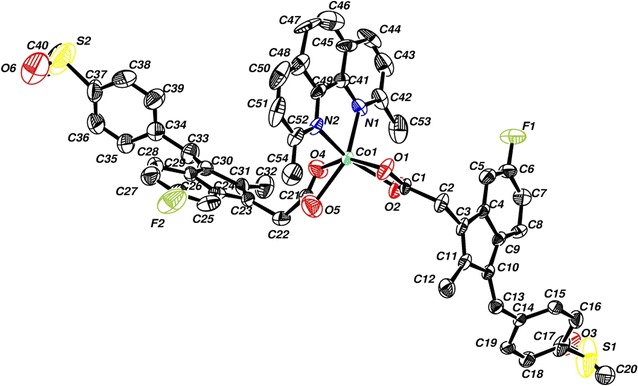
This work reports synthesis, crystallographic, spectroscopic studies and biological activity of new cobalt(II) complexes with bioactive mixed sulindac and nitrogen-donor ligands. The crystal structures of complexes** 1** and** 4** were determined using single-crystal X-ray diffraction. In-vitro anti-bacterial activity of the prepared complexes and their parent ligands were investigated against different Gram-positive and Gram-negative bacteria using agar diffusion method

## Background

Cobalt has a significant role in proteins; there are at least eight cobalt-dependent proteins. Moreover, cobalt is needed at the active center of certain coenzymes that are called cobalamins especially cyanocobalamins (Vitamin B_12_) which regulates indirectly the synthesis of DNA [1–3].

The first reported study about the biological activity of cobalt compounds was in 1952, where cobalt(III) compounds of bidentate mustard seemed to act as hypoxia-selective agents [4, 5]. Several compounds showed considerable activity against bacteria strains and against leukemia and lymphoma cell lines [6]. Furthermore, cobalt complexes possess in vivo insulin-like properties [7, 8], anti-fungal and anti-oxidant activities [9]. Several Co(III) complexes with anti-microbial activities have been reported [10–14]. For instance, a Co(III) complex of the known anti-ulcer drug famotidine turned out to have greater anti-microbial activity against *M. lysodeikticus and Escherichia coli* than the metal free drug [10–14].

Recently, metal(II) carboxylate compounds with nitrogen and/or oxygen-donor ligands have attracted an increasing interest because of their potential biological and chemical activities [15]. The interaction between heterocyclic compounds and metal ions is very important in biological systems such as drugs and vitamins [16]. In previous studies cobalt(II) compounds showed anti-fungal and anti-microbial activities; for example, imidazole-2-carbaldehyde semicarbazone was active against yeasts *Candida tropicalis* and *Saccharomyces cerevisiae*. Activity was most noticeable against phytopathogenic fungi such as Alternaria or Sclerotinia [17].

**Fig. 1:**
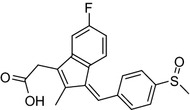
Sulindac structure [37]

{(1Z)-5-fluoro-2-methyl-1-[4-(methylsulfinyl)benzylidene]-1H-indene-3-yl}acetic acid known as Sulindac, in the form of potassium salt has a wide spectrum of activity as non-steroidal anti-inflammatory drug (NSAIDs). The chemical classes of NSAIDs comprise phenylalkanoic acids, anthranilic acids, salicylate derivatives, oxicams, furanones and sulfonamides [18–24]. Sulindac belong to phenylalkanoic acids that are potent NSAIDs for the treatment of inflammatory conditions, such as pain, fever and inflammation. The transition metal coordination with NSAIDs caused many enhanced anti-inflammatory activity [25–27]. Some compounds of NSAIDs that can coordinate with transition metals have been synthesized and tested for their biological and pharmacological activity [28–34], to our best knowledge the synthesized cobalt complexes are the first reported structures, in addition to our previously reported zinc (Fig. 1) sulindac complexes [34].

The synthesis, characterization and anti-bacterial activity of new cobalt(II) sulindac containing complexes with heterocyclic nitrogen based ligands (2-aminopyridine “2-ampy”, 1,10-phenanthroline “1,10-phen” and 2,9-dimethyl-1,10-phenanthroline “2,9-dimphen”) are described in the present work. The crystal structures of [Co(H_2_O)_4_(sul)_2_] (**1**) and [Co(2,9-dimephen)(sul)_2_] (**4**) are also reported.

## Results and discussion

### Synthesis of cobalt complexes

**Scheme 1:**
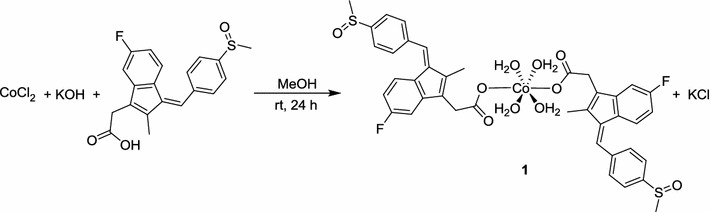
Synthesis of complex **1**

**Scheme 2:**
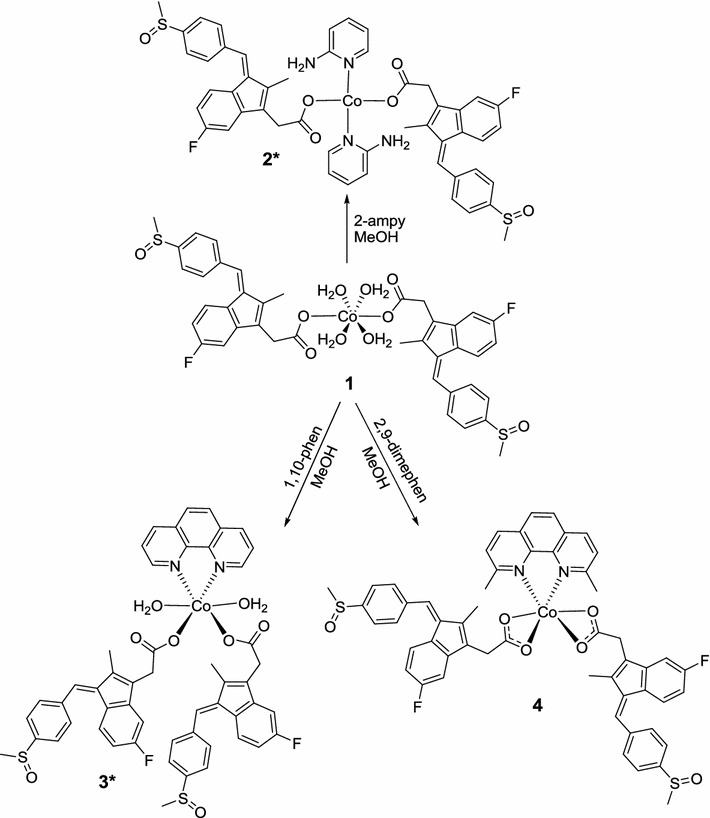
Synthesis and the proposed structures of complexes **2**–**4** (*Asterisk* proposed structure)

[Cobalt sulindac complex], **1** was prepared by mixing cobalt chloride and potassium sulindac in 1:2 molar ratios with methanol as a solvent. The desired product was obtained as a yellow solid (Scheme 1) and its structure was determined by single crystal X-ray diffraction. The novel mixed ligand cobalt(II) complexes were prepared by adding the appropriate N-donor ligand to complex **1** see (Scheme 2). The physical properties of **1–4** are summarized in Additional file 1: Table S1. Physical properties and yield of Cobalt(II) sulindac compounds.

### Crystallographic study

#### Crystallographic study of complex **1**

**Fig. 2:**
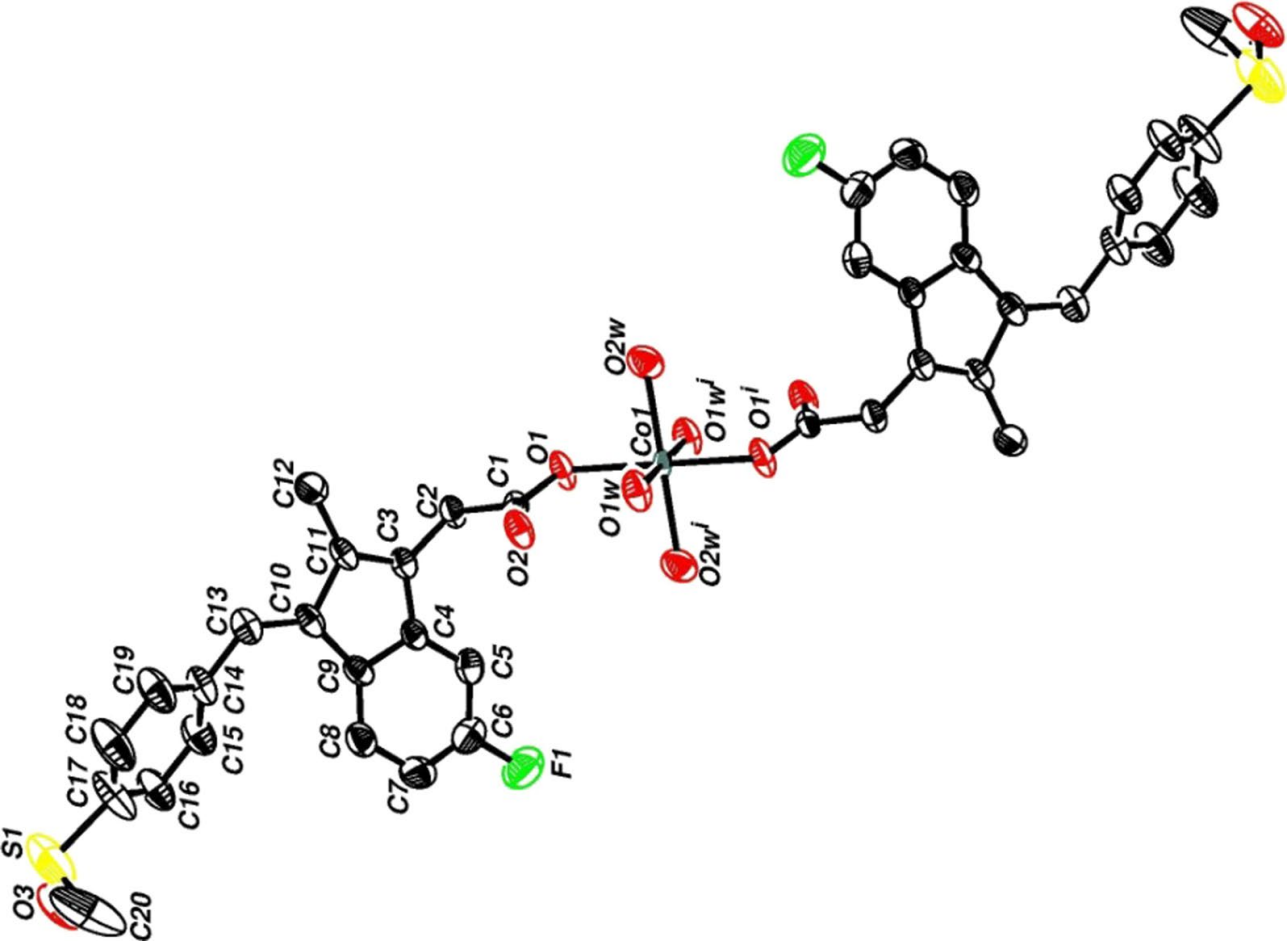
The molecular structure view of **1** showing the atom labeling scheme

The atomic numbering scheme and atom connectivity for complex **1** are shown in Fig. 2. The asymmetric unit of the titled complex, contains a Co(II) cation, two monodentate sulindac groups and four water molecules.

Although the synthetic procedure and the recrystallization process of complex **1** were performed in methanol, a marked preference for coordination of water over methanol was observed and proved by single crystal X-ray determination. This phenomenon might be due to the stronger bond interaction between water and the metal center than methanol. In addition, the used methanol was not dry enough and wet, so it was possible to provide the four water molecules bonded to the metal center.

The two sulindaco groups are connected to the metal center in a monodentate coordination mode forming a symmetrical octahedral geometry with the additional four water molecules. The Co–O bond distances of 2.089(4), 2.100(5) and 2.141(4) Å are similar to previously reported values [38]. Selected bond angles and bond distances are listed in Table 1.

**Table 1:** Selected bond angles (°) and bond distances (Å) for **1** and **4**

Bond distance (Å) of complex **1**		Bond distance (Å) of complex **4**	
Co(1)–O(1)	2.089(4)	Co(1)–O(1)	2.133(7)
Co(1)–O(1)#1	2.089(4)	Co(1)–O(4)	2.117(8)
Co(1)–O(2W)#1	2.100(5)	Co(1)–O(2)	2.128(6)
Co(1)–O(2W)	2.100(5)	Co(1)–O(5)	2.220(10)
Co(1)–O(1W)	2.141(4)	Co(1)–N(1)	2.100(7)
Co(1)–O(1W)#1	2.141(4)	Co(1)–N(2)	2.090(7)

Bond angle (°) of complex **1**		Bond angle (°) of complex **4**	
C(1)–O(1)–Co(1)	126.2(4)	C(1)–O(2)–Co(1)	91.5(5)
O(1 W)–Co(1)–O(1W)#1	180.000(2)	C(21)–O(4)–Co(1)	94.2(7)
O(2 W)–Co(1)–O(2W)#1	180.000(1)	C(21)–O(5)–Co(1)	87.6(7)
O(1)–Co(1)–O(1)#1	180.000(2)	N(2)–Co(1)–N(1)	79.8(3)
O(1)#1–Co(1)–O(2W)#1	87.9(2)	N(1)–Co(1)–O(1)	108.2(3)
O(1)–Co(1)–O(2W)	87.9(2)	N(2)–Co(1)–O(4)	112.3(3)
O(1)–Co(1)–O(1W)	92.09(17)	N(1)–Co(1)–O(2)	104.3(3)
O(1)#1–Co(1)–O(1W)	87.91(17)	N(2)–Co(1)–O(5)	102.5(4)
O(2W)#1–Co(1)–O(1W)	89.4(2)	O(4)–Co(1)–O(1)	155.4(3)
		O(2)–Co(1)–O(5)	91.9(4)
		O(2)–Co(1)–O(1)	59.1(3)
		C(1)–O(1)–Co(1)	90.7(5)

From the bonding angles in complex **1**; O(1)#1–Co(1)–O(2W)#1 = 87.9(2)°, O(1)–Co(1)–O(2W) = 87.9(2)°, O(1)–Co(1)–O(1 W) = 92.09(17)°, O(1)#1–Co(1)–O(1W) = 87.91(17)° and O(2W)#1–Co(1)–O(1W) = 89.4(2)° a slight distortion from regular octahedral geometry was observed due to the expected *Jahn*–*Teller* effect which is also confirmed by the appearance of a shoulder in the *d*–*d* visible transition of this and other cobalt complexes.

#### Crystallographic study of complex **4**

**Fig. 3:**
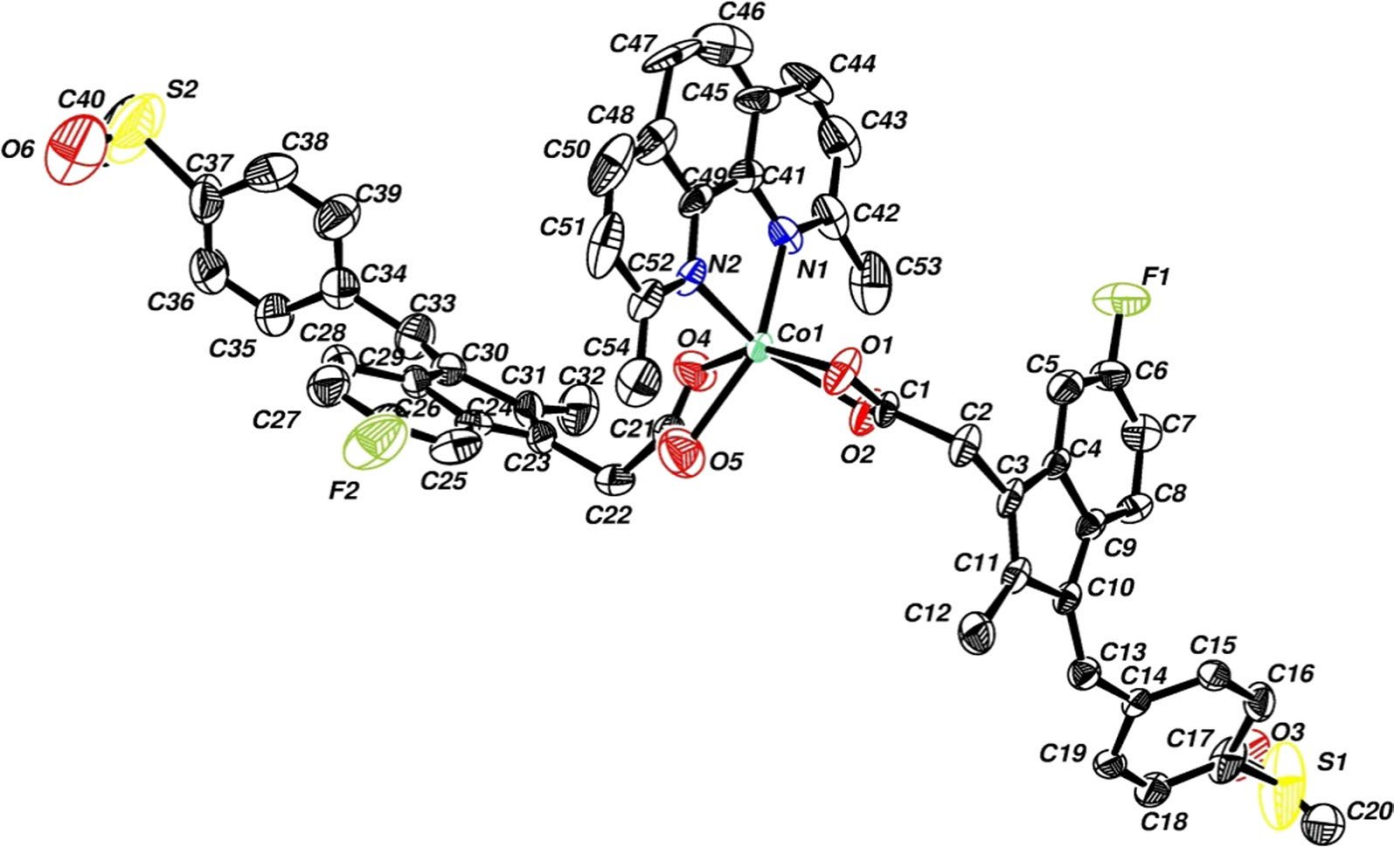
The molecular structure view of **4** showing the atom labeling scheme

The atomic numbering scheme and atom connectivity for complex **4** are shown in Fig. 3. The asymmetric unit of the titled complex, contains a Co(II) cation, two sulindac groups and one 2,9-dimephen ligand. The Co–O bond distances of 2.117(8), 2.128(6), 2.220(10) and 2.220(10) Å are similar to reported values [39–47]. Co–N bond distances of 2.090(7) and 2.100(7) Å are also similar to reported values [39–48]. Selected angles and distances are listed in Table 1.

From bonding angles in complex **4**, a slight deviation from octahedral geometry was observed, N(1)–Co(1)–O(1) = 108.2(3)°, N(2)–Co(1)–O(4) = 112.3(3)°, N(2)–Co(1)–O(5) = 102.5(4)°, N(2)–Co(1)–N(1) = 79.8(3)° and N(1)–Co(1)–O(2) = 104.3 (19)°.

### Infrared spectra

Infrared spectral data of KBr pellet of cobalt sulindac complexes **1–4** in the 400–4000 cm^−1^ range are summarized in Additional files 2 and 3: Table S2. Comparison between some of principle peaks in IR for K(sul) and **1** (cm^-1^) and Table S3. Summary of principle peaks in IR for complexes **2**, ** 3** and **4** (cm^-1^). In metal carboxylate complexes, the major characteristic of the IR spectra is the frequency of the υ asymmetric (υ_as_) and υ symmetric (υ_s_) of carbonyl (COO^−^) stretching vibrations and the difference between them Δυ(COO^−^). The frequency of these bands depends upon the coordination mode of the carboxylate ligand. Monodentate complexes exhibit Δυ(COO^−^) values that are much greater than the ionic complexes. Chelating (bidentate) complexes exhibit Δυ(COO^−^) values that are significantly less than the ionic values. Δυ(COO^−^) values for bridging complexes are greater than those of chelating complexes, and close to the ionic values [49]. In complex **1**; υ_as_(COO^−^) is at 1601 cm^−1^ and υ_s_(COO^−^) at 1397 cm^−1^, Δυ(COO^−^) = 204 cm^−1^ which is close to that of potassium sulindac which supports a coordination mode for complex **1** as monodentate. The O–H vibration frequency at 3376 cm^−1^ indicates the presence of water molecules in the coordination geometry [Co(H_2_O)_4_(sul)_2_] as also supported by single crystal X-ray determination.

The assignments of IR frequencies for the asymmetric stretching υ_as_(COO^−^), the symmetric stretching υ_s_(COO^−^) and the difference between these two values of sulindac group in complexes **1**–**4** and those of potassium sulindac are shown in Additional file 1: Tables S2 and S3.

Complexes **2** and **3** have υ_as_(COO^−^) at 1599, and 1600 cm^−1^, but υ_s_(COO^−^) appear at 1390 and 1380 cm^−1^, so Δυ (COO^−^) are 219 and 220 cm^−1^, respectively which is larger than Δυ(COO^−^)_K(sul)_ = 178 cm^−1^ and this supports monodentate coordination mode of the carboxylate groups. In addition, complex **3** has an absorption frequency at 3415 cm^−1^ which may indicate water molecules in the coordination geometry.

Moreover, in complex **2** two absorption frequencies υ_as_(NH_2_) at 3374 cm^−1^ and υ_s_(NH_2_) at 3268 cm^−1^ with Δυ(NH_2_) = 106 cm^−1^ were observed. These frequencies are assigned to the 1°-NH_2_ group indicating that the complexation with cobalt is through the pyridine nitrogen atom rather than the NH_2_ nitrogen atom [50, 51].

In complex **4** υ_as_(COO^−^) was observed at 1599 cm^−1^, and υ_s_(COO^−^) was at 1441 cm^−1^ giving a Δυ(COO^−^) of 158 cm^−1^ and this supports a bidentate coordination mode of the carboxylate groups. This result was also confirmed by X-ray structure determination of complex **4**.

### UV–Vis spectra

Generally, three types of electronic transitions have been observed for coordination compounds: Metal to ligand (MLCT) or ligand to metal (LMCT) charge-transfer absorption bands, *d*–*d* transition bands and intra-ligand (LC) transition bands [52, 53].

Co(II) metal ion with low spin *d* ^*7*^ electronic configuration showed two low intensity bands with small *ε* value (12–13 Lmol^−1^ cm^−1^) in the visible region. The source of these two bands is due to the *d*–*d* transition between ^2^E^2^→T_1g_ and ^2^E→^2^T_2g_. LMCT was observed at (206–213 nm) with *ε* values between 1800 and 3000 Lmol^−1^ cm^−1^ [20, 21, 54–67]. All other bands are similar to nitrogen based ligand *Π*→Π* or n→Π* transitions with small blue or red shifts for cobalt coordination complexes [20, 21, 55–67]. The results are tabulated in Additional file 4: Table S4. UV-visible spectral data for compounds (**1**–**4**).

Complexes **3** and **4** adopted distorted octahedral geometries with different carboxylate coordination modes, e.g. monodentate, bidentate, in complex **3** the two water molecules were covalently coordinated to the central Co(II) cation which imposed monodentate coordination mode of the sulindaco groups. Whereas, the two sulindaco groups in complex **4** are both bidentately coordinated to the Co(II) center as a result of the increased steric hindrance effect by two methyl groups on the 1,10-phen ring. The electronic effect of the ligands in complexes **2–4** are almost identical.

### Magnetic properties

The magnetic moment measurements of compounds **1**–**4** are given in Table 2. The value of magnetic moments for all complexes indicates that each compound has paramagnetic properties with one unpaired electron, which indicates that each Co(II) complex adopted a low spin, *d* ^*7*^ octhedral geometry. Low spin Co(II) octahedral complexes with nitrogen and/or oxygen-donor ligands are very rare [62]. Both structural, magnetic and spectral data are necessary to prove that a complex contains low spin Co(II) metal ion octahedral geometry with only few of these compounds have been structurally characterized by single crystal X-ray crystallography [68–71].

**Table 2:** Magnetic properties of cobalt(II) compounds

Compounds	Magnetic moment (μ_eff_ BM)	Unpaired electron (n)
[Co(H_2_O)_4_(sul)_2_] (**1**)	2.26 ± 0.05	1
[Co(2-ampy)_2_(sul)_2_] (**2**)	2.41 ± 0.15	1
[Co(H_2_O)_2_(1,10-phen)(sul)_2_] (**3**)	2.40 ± 0.12	1
[Co(2,9-dimephen)(sul)_2_] (**4**)	2.40 ± 0.09	1

### Anti-bacterial activity

Before measurement of their biological activity, the solution stability of the complexes were tested, as the complexes were crystallized by slow solvent evaporation at room temperature that took several days and the same physical properties of the compounds were obtained. Moreover, the relevant X-ray structure determination of some complexes showed that the structures were remained intact.

Two Gram positive bacteria (*Staphylococcus epidermidis, Staphylococcus aureus*), two Gram negative bacteria (*Bordetella, E. coli*) and yeast species (*Saccharomyces and Candida*) were used to test the compounds anti-bacterial activity. The results were obtained by the well-diffusion method using DMSO as a negative control to resist any tested microorganisms; Gentamycin as a positive control for Gram positive and Gram negative bacteria and Nystatin as a positive control for yeast. The parent ligand, potassium sulindac, did not show anti-bacterial activity against any of the tested microorganisms, but (CoCl_2_) showed anti-bacterial activity against all tested microorganisms (Table 3).

**Table 3:** In-vitro anti-bacterial activity data of complexes **1**–**4**

Compounds	*Bordetella*	*E. coli*	*S. epi*	*S. aureus*	*Candida*	*Saccharomyces*
	G-	G-	G+	G+	Yeast	Yeast
(**1**)	15.3 ± 0.5	10.1 ± 0.4	21.0 ± 0.4	19 ± 1	–	–
(**2**)	13 ± 1	–	23 ± 1	11 ± 1	–	–
(**3**)	12 ± 2	8.5 ± 1.5	26.7 ± 0.6	21 ± 1	–	–
(**4**)	16 ± 2	12 ± 2	39 ± 1	25.0 ± 1.5	42 ± 1	41.12 ± 0.5
CoCl_2_	22 ± 2	12 ± 2	30.0 ± 0.5	11 ± 1	20.0 ± 0.7	22 ± 1
Sulindac	–	–	–	–	–	–
Genta.	30 ± 1	37 ± 1	28 ± 1	32.7 ± 0.6	–	–
Nes.	–	–	–	–	35.5 ± 0.2	40.5 ± 0.4

Inhibition zone diameter (IZD) in mm, all microorganisms were resistant to DMSO. The data stated as average ± standard deviation (N = 3), the concentration of the complexes and the standards was 30 mg/5 mL in DMSO (6 g/l)

— dashes indicated zero inhibition

Complex **1** showed high activity against G^−^ or G^+^ bacteria except against *E. coli*. Complexes **3** and **4** showed low activity against G^−^ bacteria and high activity against G^+^ bacteria. Complex **2** showed high activity against *S. epidermidis* and low or zero activity against other bacteria. However, in yeast all complexes didn’t show any activity except complexes **4** showed high activity. Complexes **3** and **4** were chosen for further studies because of their higher IZD values. The complexes have been studied with their parent nitrogen donor ligands “1,10-phen and 2,9-dimephen” against all tested Gram-positive, Gram-negative bacteria and yeast to determine the effect of the complexation on anti-bacteria activity (Tables 4, 5).

**Table 4:** Comparison of anti-bacterial activity of complex **3** with **1**,**10**-**phen**

Concentration (mg/ml)	*Bordetella*	*E. coli*	*S. epidermidis*	*S. aureus*
	G−	G−	G+	G+
IZD of **3** (mm)				
**8**	11.9 ± 2	8.5 ± 1.5	26.7 ± 0.6	21 ± 1
**4**	10.3 ± 0.5	–	24.6 ± 1.5	18.7 ± 0.5
**2**	–	–	22.6 ± 1.6	10.9 ± 0.7
IZD of **1**,**10**-**phen**				
**8**	33.0 ± 0.7	33 ± 1	36 ± 0.6	38.5 ± 1.5
**4**	21.6 ± 0.5	31.5 ± 1.7	33.6 ± 0.7	35.4 ± 0.5
**2**	11.0 ± 1	29.0 ± 0.7	24 ± 1.6	28.6 ± 0.7

**Table 5:** Comparison of anti-bacterial activity of complex **4** with **2**,**9**-**dimephen**

Concentration (mg/ml)	*Bordetella*	*E. coli*	*S. epidermidis*	*S. aureus*	*Saccharomyces*
	G−	G−	G+	G+	Yeast
IZD of **4** (mm)					
**8**	16.2 ± 1.9	12.0 ± 2.0	39 ± 1	25.0 ± 1.5	41.12 ± 0.5
**4**	13.7 ± 0.5	–	34.6 ± 0.7	24.3 ± 0.5	41 ± 1
**2**	11.4 ± 1.2	–	30.4 ± 1.6	21.9 ± 0.7	35.9 ± 0.5
IZD of **2**, **9**-**dimephen**					
**8**	14.6 ± 0.9	–	36.9 ± 1.5	39 ± 1	44 ± 2
**4**	9.2 ± 0.5	–	35.5 ± 0.7	35.4 ± 0.5	42 ± 1
**2**	8.3 ± 1.2	–	33.0 ± 1.6	31.3 ± 0.7	38.4 ± 0.5

Tables 4 and 5 show that the complexation process of cobalt-sulindac with 1,10-phen in complex **3** decreased the anti-bacterial activity considerably for both gram negative and gram positive bacteria, but complexation of cobalt-sulindac with 2,9-dimephen in complex **4** mostly showed similar behavior against *S. epidermidis* and yeast, but decreased the activity against *S. aureus* and increased the anti-bacterial activity against gram negative bacteria. The anti-bacterial activity of complexes **1–4** when compared with previously reported work would be considered as promising results [15, 28–36, 72–78].

## Conclusion

Four new Co(II) complexes with sulindac in the presence of N-donor heterocyclic ligands (2-ampy, 1,10-phen and 2,9-dimephen) have been synthesized and characterized. Magnetic properties, infrared and UV–Vis spectrophotometric techniques were used to study the new complexes in addition to X-ray diffraction of complexes **1** and **4**; which reveals distorted octahedral geometry of the Co(II) ion. In complex **1** the cobalt binds two monodentate sulindac groups and in complex **4** cobalt binds two bidentate sulindac groups and one 2,9-dimephen. The structures of the remaining complexes were proposed depending on IR, UV–Vis results and magnetic properties. Complexes **3** and **4** showed anti-bacterial activity against G^+^ and G^−^ bacteria. Moreover, complex **4** have demonstrated the highest efficiency against yeast.

The results of this work was Submitted in Partial Fulfillment of the Requirements for the Degree of Masters in Applied Chemistry, Faculty of Graduate Studies, Birzeit University, Ramallah, Palestine. The thesis was published in 2015 on FADA Birzeit University Open Access Repository [79].

## Experimental

### Starting materials

Cobalt(II) chloride was purchased from Merck, sulindac, 2-aminopyridine, 1,10-phenanthroline and 2,9-dimethyl-1,10-phenanthroline were purchased from Sigma-Aldrich. All solvents used were of analytical reagent grade and purchased from commercial sources. *E. coli, S. aureus, S. epidermidis, Bordetella* and Yeast species (*Saccharomyces and candida*) were kindly obtained from the Drugs Department at Central Public Health Laboratory.

### Synthesis

All Co(II) complexes were synthesized at room temperature in ambient conditions.

#### Synthesis of **[Co(H**_**2**_**O)**_**4**_**(sul)**_**2**_**]** (**1**)

Sulindac (3.0 g, 8.4 mmol) was allowed to dissolve in a methanolic solution of potassium hydroxide (0.47 g, 4.2 mmol) (75 ml methanol). To this solution was added slowly CoCl_2_·7H_2_O (1.0 g, 4.2 mmol) in 15 ml of methanol. The mixture was allowed to stir for 24 h and the formed precipitate was collected, washed with cold water and air dried. Suitable crystals for X-ray structural analysis were obtained by recrystallization from hot methanol.

**[Co(H**_**2**_ **O)**_**4**_ **(sul)**_**2**_ **]** (**1**): 85% (3.81 g) yield; m.p. 201 °C; IR (cm^−1^, KBr): 3376, 3050, 2911, 2850, 1600, 1563, 1485, 1465, 1416, 1369, 1326,1268, 1217, 1203, 1171, 1133, 1086, 1024, 1008, 967, 918, 891, 891, 868, 805, 776, 717, 672, 659, 572, 473; UV–Vis [DMSO, λ (nm)(є/Lmol^−1^ cm^−1^)]: 211 (3283), 252 (828), 258 (872), 264 (850), 282 (771), 328 (514); μ_eff_ = 2.26 BM.

#### Synthesis of **[Co(2-ampy)**_**2**_**(Sul)**_**2**_**]** (**2**)

Sulindac (3.0 g, 8.4 mmol) was allowed to dissolve in a methanolic solution of potassium hydroxide (0.47 g, 4.2 mmol) (40 ml methanol). To this solution was added slowly CoCl_2_·7H_2_O (1.0 g, 4.2 mmol) in 10 ml of methanol, then 2-ampy (0.79 g, 8.4 mmol) dissolved in 15 ml of methanol was added. The mixture was allowed to stir for 24 h, the solvent was evaporated then the residue was dissolved in dichloromethane which was then evaporated and the compound obtained was washed with petroleum ether and dried under vacuum.

**[Co(2-ampy)**_**2**_ **(Sul)**_**2**_ **]** (**2**): 56% (2.50 g) yield; m.p. 180 °C (decomposed); IR (cm^−1^, KBr): 3374, 3268, 3015, 2914, 2860, 1599, 1515, 1494, 1464, 1424, 1380, 1267, 1195, 1164, 1137, 1086, 1031, 1010, 955, 915, 891, 846, 811, 727, 651, 593, 533, 474, 449; UV–Vis [DMSO, λ (nm); (є/Lmol^−1^ cm^−1^)]: 207 (1828), 286 (450), 329 (348), 655 (12.7); μ_eff_ = 2.41 BM.

#### Synthesis of **[Co(H**_**2**_**O)**_**2**_**(1,10-phen)(sul)**_**2**_**]** (**3**)

Sulindac (3.0 g, 8.4 mmol) was allowed to dissolve in a methanolic solution of potassium hydroxide (0.47 g, 4.2 mmol) (40 ml methanol). To this solution was added slowly CoCl_2_·7H_2_O (1.0 g, 4.2 mmol) in 10 ml of methanol, then 1,10-phenanthroline (0.756 g, 4.2 mmol) dissolved in 15 ml of methanol was added. The mixture was allowed to stir for 24 h, the solvent was evaporated then the residue was dissolved in dichloromethane which was then evaporated and the compound obtained was washed with petroleum ether and dried under vacuum.

**[Co(H**_**2**_ **O)**_**2**_ **(1,10-phen)(sul)**_**2**_ **]** (**3**): 22% (1.0 g) yield; m.p. 140 °C; IR (cm^−1^, KBr): 3415, 3059, 2911, 2852, 1600, 1515, 1464, 1424, 1380, 1267, 1195, 1164, 1137, 1086, 1010, 956, 915, 891, 846, 811, 727, 651, 593, 533, 474, 441; UV–Vis [DMSO, λ (nm) (є/Lmol^−1^ cm^−1^)]: 208 (2152), 226 (700), 271 (535), 328 (224), 431 (16.3), 488 (13.2); μ_eff_ = 2.4 BM.

#### Synthesis of **[Co(2,9-dimephen)(sul)**_**2**_**]** (**4**)

Sulindac (3.0 g, 8.4 mmol) was allowed to dissolve in a methanolic solution of potassium hydroxide (0.47 g, 4.2 mmol) (40 ml methanol). To this solution was added slowly CoCl_2_·7H_2_O (1.0 g, 4.2 mmol) in 10 ml of methanol, then 2,9-dimethyl-1,10-phenanthroline (0.875 g, 4.2 mmol) dissolved in 15 ml of methanol was added. The mixture was allowed to stir for 24 h, the solvent was evaporated then the residue was dissolved in dichloromethane which was then evaporated and the compound obtained was washed with petroleum ether and dried. Suitable crystals for X-ray structural analysis were obtained by recrystallization from 1:1 mixture of chloroform/acetonitrile.

**[Co(2,9-dimephen)(sul)**_**2**_ **]** (**4**): 34% (1.54 g) yield; m.p. 150 °C (decomposed); IR (cm^−1^, KBr): 3040, 2912, 2845, 1599, 1566, 1465, 1441, 1359, 1194, 1157, 1135, 1086, 1031, 954, 916, 891, 855, 812, 761, 728, 644, 533, 474; UV–Vis [DMSO, λ (nm) (є/Lmol^−1^ cm^−1^)]: 207 (2263), 229 (933), 274 (621), 328 (261), 432 (13.3); μ_eff_ = 2.4 BM.

### Physical measurements

Infrared (IR) spectra were recorded in the 450–4000 cm^−1^ region (KBr) on a Perkin Elmer FT-IR spectrometer (2004). UV–Vis spectra were recorded using Hewlett Packard 8453 photo diode array spectrophotometer in the 200–800 nm region using DMSO as solvent. Melting points were determined in capillary tubes with B-545 melt apparatus without any correction. The magnetic susceptibility measurements were determined by Gouy method using mercury cobalt-thiocyanate complex, (HgCo(NSC)_4_) as standard. Calculation of the effective magnetic moment was obtained by using the following: μ_eff_ = 2.83 * (χ_m_T)^1/2^ (Molar susceptibility, χ_m_, and T is the temperature with K).

#### X-ray crystallography

X-ray intensity data of complexes **1** and **4** was carried out at room temperature on a Bruker SMART APEX CCD X-ray diffractometer system (graphite-monochromated Mo Kα radiation λ = 0.71073 Å) by using the SMART software package [80]. The data were reduced and integrated by the SAINT program package [81]. The structure was solved and refined by the SHELXTL software package [82]. H atoms were located geometrically and treated with a riding model. The R-factor above 10% reflects the low quality of crystals obtained in the process of recrystallization and better crystals could not been found. Crystal data and details of the data collection and refinement are summarized in Table 6 and in Additional file 5: Supplementary crystallographic data for complexes **1** and **4**.

**Table 6:** Structure refinement of crystal data for compounds (**1**) and (**4**)

	Complex (**1**)		Complex (**4**)	
Empirical formula	C_40_ H_34_CoF_2_O_12_S_2_		C_53_ H_38_CoF_2_N_2_O_5_S_2_	
Formula weight	867.72		943.90	
Wavelength	0.71073 Å		0.71073 Å	
Temperature	295(1) K		295(1) K	
Space group	P-1		P2(1)/c	
Crystal system	Triclinic		Monoclinic	
Unit cell dimensions	a = 5.012(3) Å	α = 81.85(1)°	a = 20.930(3) Å	α = 90°
	b = 12.640(8) Å	β = 82.230(9)°	b = 14.836(2) Å	β = 101.705°
	c = 16.22(1) Å	γ = 86.40(1)°	c = 15.807(2) Å	γ = 90°
Volume	1006.9(11) Å^3^		4806.3(11) Å^3^	
Z	1		4	
Absorption coefficient	0.601 mm^−1^		0.500 mm^−1^	
Density (calculated)	1.431 Mg/m^3^		1.304 Mg/m^3^	
Crystal size	0.50 × 0.16 × 0.06 mm^3^		0.53 × 0.46 × 0.05 mm^3^	
F(000)	447		1948	
Reflections collected	10,787		52,864	
Theta range for data collection	2.56–27.00°		1.69–27.00°	
Index ranges	−6 ≤ h ≤ 6, −16 ≤ k ≤ 16, −20 ≤ l ≤ 20		−26 ≤ h ≤ 26, −18 ≤ k ≤ 18, −20 ≤ l ≤ 19	
Completeness to theta = 26.99°	98.5%		99.7%	
Independent reflections	4334[R(int) = 0.0625]		10,468 [R(int) = 0.0766]	
Absorption correction	None		None	
Data/restraints/parameters	4334/0/273		10,468/0/603	
Refinement method	Full-matrix least-squares on F^2^		Full-matrix least-squares on F^2^	
Largest diff. peak and hole	1.331 and −0.664 e Å^−3^		2.147 and −0.686 e Å^−3^	
Goodness-of-fit on F^2^	1.210		1.576	
R indices (all data)	R1 = 0.1355, wR^2^ = 0.2727		R1 = 0.2349, wR^2^ = 0.4718	
Final R indices^a^ [I > 2sigma(I)]	R1 = 0.1158, wR^2^ = 0.2599		R1 = 0.1941, wR^2^ = 0.4496	

^a^ $${\text{R}}1 = \sum \left\| {{\text{F}}_{0} \left| - \right|{\text{F}}_{\text{c}} } \right\|/\sum {\text{F}}_{0} ,\;{\text{wR}}_{2} = \left\{ {\sum [{\text{w}}({\text{F}}_{0}^{2} - {\text{F}}_{c}^{2} )^{2} ]/\sum [{\text{w}}({\text{F}}_{0}^{2} )^{2} } \right\}^{1/2}$$

### Anti-bacterial activity

Agar diffusion method [83] was used for screening the anti-bacterial activity measurements of the synthesized cobalt complexes. Different types of gram-negative bacteria (*Bordetella, E. coli*) and gram-positive (*S. epidermidis, S. aureus*) and Yeast species (*Saccharomyces and Candida*) were used in the present work.

In sterile saline single bacterial colonies were dissolved until the suspended cells reached the turbidity of McFarland 0.5 Standard. The bacterial inocula were spread on the surface of the Muller Hinton nutrient agar by means of a sterile cotton swab. Sterile glassy borer were used to make a 6 mm in diameter wells in the agar plate. Samples were dissolved in DMSO in concentration equal to (8 mg/ml), (4 mg/ml) and (2 mg/ml), then 50 μl of the test samples were introduced in the respective wells. DMSO was used as negative control while gentamycin used as positive control. Immediately the plate was incubated at 37 °C for 24 h. The anti-bacterial activity was determined by measuring the diameter inhibition zone of complete growth in millimeter (mm). The averages of two trials determined the results and are stated as average ± standard deviation.

## Additional files

**Additional file 1: Table S1.** Physical properties and yield of Cobalt(II) sulindac compounds.

**Additional file 2: Table S2.** Comparison between some of principle peaks in IR for K(sul) and 1 (cm^-1^).

**Additional file 3: Table S3.** Summary of principle peaks in IR for complexes 2, 3 and 4 (cm^-1^).

**Additional file 4: Table S4.** UV-visible spectral data for compounds (**1**–**4**).

**Additional file 5:** CCDC 1450310 and CCDC 1450311 contain the supplementary crystallographic data for complexes 1 and 4. These data can be obtained free of charge via http://www.ccdc.cam.ac.uk/conts/retrieving.html, or from the Cambridge Crystallographic Data Centre, 12 Union Road, Cambridge CB2 1EZ, UK; fax: (+44) 1223-336-033; or e-mail: deposit@ccdc.cam.ac.uk. Supplementary data associated with this article can be found, in the online version.
