# Supplementary material for: Synthesis, crystallographic, spectroscopic studies and biological activity of new cobalt(II) complexes with bioactive mixed sulindac and nitrogen-donor ligands
Source: Chem Cent J. 2017 May 10;11:40. doi: 10.1186/s13065-017-0268-2 (PMC5423883; doi:10.1186/s13065-017-0268-2)

checkCIF/PLATON report

Structure factors have been supplied for datablock(s) hijazi3m

THIS REPORT IS FOR GUIDANCE ONLY. IF USED AS PART OF A REVIEW PROCEDURE FOR PUBLICATION, IT SHOULD NOT REPLACE THE EXPERTISE OF AN EXPERIENCED CRYSTALLOGRAPHIC REFEREE.

No syntax errors found. CIF dictionary Interpreting this report

Datablock: hijazi3m

```
Bond precision:  C-C = 0.0103 A          Wavelength=0.71073

Cell:          a=5.012(3)         b=12.640(8)      c=16.22(1)
               alpha=81.852(10)   beta=82.230(9)   gamma=86.395(10)
Temperature:   295 K

                Calculated                 Reported
Volume          1006.9(11)                 1006.9(11)
Space group     P -1                       P-1
Hall group      -P 1                       -P 1
Moiety formula  C40 H34 Co F2 O10 S2, 2(O) C40 H34 Co F2 O10 S2, 2(O)
Sum formula     C40 H34 Co F2 O12 S2       C40 H34 Co F2 O12 S2
Mr              867.72                     867.72
Dx,g cm-3       1.431                      1.431
Z               1                          1
Mu (mm-1)       0.601                      0.601
F000            447.0                      447.0
F000'           447.84
h,k,lmax        6,16,20                    6,16,20
Nref            4401                       4334
Tmin,Tmax       0.891,0.965
Tmin'           0.740

Correction method= Not given

Data completeness= 0.985          Theta(max)= 27.000

R(reflections)= 0.1158( 3560)     wR2(reflections)= 0.2727( 4334)

S = 1.210                  Npar= 273
```

The following ALERTS were generated. Each ALERT has the format
test-name_ALERT_alert-type_alert-level.
Click on the hyperlinks for more details of the test.

Alert level A

```
PLAT183_ALERT_1_A Missing _cell_measurement_reflns_used value ....     Please Do !
PLAT184_ALERT_1_A Missing _cell_measurement_theta_min value ......     Please Do !
PLAT185_ALERT_1_A Missing _cell_measurement_theta_max value ......     Please Do !
PLAT902_ALERT_1_A No (Interpretable) Reflections found in FCF ....     Please Check
```

Alert level B

```
PLAT234_ALERT_4_B Large Hirshfeld Difference S1     --  C20     ..       0.29 Ang.
PLAT306_ALERT_2_B Isolated Oxygen Atom (H-atoms Missing ?) .......        O3W Check
PLAT417_ALERT_2_B Short Inter D-H..H-D       H2W1    ..  H1W2    ..       2.04 Ang.
PLAT430_ALERT_2_B Short Inter D...A Contact  O3      ..  O3W     ..       2.82 Ang.
```

Alert level C

```
RFACG01_ALERT_3_C  The value of the R factor is > 0.10
            R factor given   0.116
RFACR01_ALERT_3_C  The value of the weighted R factor is > 0.25
            Weighted R factor given   0.273
PLAT082_ALERT_2_C High R1 Value ..................................       0.12 Report
PLAT084_ALERT_3_C High wR2 Value (i.e. > 0.25) ...................       0.27 Report
PLAT215_ALERT_3_C Disordered O3       has ADP max/min Ratio .....         3.1 Note
PLAT215_ALERT_3_C Disordered C20      has ADP max/min Ratio .....         4.0 Note
PLAT220_ALERT_2_C Non-Solvent Resd 1   C   Ueq(max)/Ueq(min) Range        4.4 Ratio
PLAT241_ALERT_2_C High   'MainMol' Ueq as Compared to Neighbors of         S1 Check
PLAT241_ALERT_2_C High   'MainMol' Ueq as Compared to Neighbors of         O1 Check
PLAT242_ALERT_2_C Low    'MainMol' Ueq as Compared to Neighbors of        Co1 Check
PLAT250_ALERT_2_C Large U3/U1 Ratio for Average U(i,j) Tensor ....        2.1 Note
PLAT341_ALERT_3_C Low Bond Precision on  C-C Bonds ...............    0.01029 Ang.
PLAT925_ALERT_1_C The Reported and Calculated Rho(max) Differ by .       1.33 eA-3
```

Alert level G

```
PLAT005_ALERT_5_G No Embedded Refinement Details found  in the CIF     Please Do !
PLAT007_ALERT_5_G Number of Unrefined Donor-H Atoms ..............          4 Report
PLAT072_ALERT_2_G SHELXL First  Parameter in WGHT  Unusually Large       0.10 Report
PLAT093_ALERT_1_G No s.u.'s on H-positions, Refinement Reported as      mixed Check
PLAT230_ALERT_2_G Hirshfeld Test Diff for    S1     --  O3      ..       16.1 s.u.
PLAT230_ALERT_2_G Hirshfeld Test Diff for    S1     --  O3A     ..        8.5 s.u.
PLAT232_ALERT_2_G Hirshfeld Test Diff (M-X)  Co1    --  O1W     ..        6.3 s.u.
PLAT300_ALERT_4_G Atom Site Occupancy of *O3     is Constrained at        0.5 Check
PLAT300_ALERT_4_G Atom Site Occupancy of *O3A    is Constrained at        0.5 Check
PLAT300_ALERT_4_G Atom Site Occupancy of *C20    is Constrained at        0.5 Check
PLAT300_ALERT_4_G Atom Site Occupancy of *C20A   is Constrained at        0.5 Check
PLAT301_ALERT_3_G Main Residue  Disorder ............ Percentage =          7 Note
PLAT432_ALERT_2_G Short Inter X...Y Contact  O1W    ..  C20A    ..       2.95 Ang.
PLAT432_ALERT_2_G Short Inter X...Y Contact  O2W    ..  C20A    ..       2.81 Ang.
PLAT432_ALERT_2_G Short Inter X...Y Contact  O3W    ..  C20A    ..       2.66 Ang.
PLAT720_ALERT_4_G Number of Unusual/Non-Standard Labels ..........          4 Note
PLAT779_ALERT_4_G Suspect or Irrelevant (Bond) Angle in CIF .... #         54 Check
             O3A  -S1   -C20     1.555   1.555   1.555             39.20 Deg.
PLAT899_ALERT_4_G SHELXL97   is Deprecated and Succeeded by SHELXL       2014 Note
```

```
   4 ALERT level A = Most likely a serious problem - resolve or explain
   4 ALERT level B = A potentially serious problem, consider carefully
  13 ALERT level C = Check. Ensure it is not caused by an omission or oversight
  18 ALERT level G = General information/check it is not something unexpected

   6 ALERT type 1 CIF construction/syntax error, inconsistent or missing data
```

```
 16 ALERT type 2 Indicator that the structure model may be wrong or deficient
  7 ALERT type 3 Indicator that the structure quality may be low
  8 ALERT type 4 Improvement, methodology, query or suggestion
  2 ALERT type 5 Informative message, check
```

It is advisable to attempt to resolve as many as possible of the alerts in all categories. Often the minor alerts point to easily fixed oversights, errors and omissions in your CIF or refinement strategy, so attention to these fine details can be worthwhile. In order to resolve some of the more serious problems it may be necessary to carry out additional measurements or structure refinements. However, the purpose of your study may justify the reported deviations and the more serious of these should normally be commented upon in the discussion or experimental section of a paper or in the "special_details" fields of the CIF. checkCIF was carefully designed to identify outliers and unusual parameters, but every test has its limitations and alerts that are not important in a particular case may appear. Conversely, the absence of alerts does not guarantee there are no aspects of the results needing attention. It is up to the individual to critically assess their own results and, if necessary, seek expert advice.

Publication of your CIF in IUCr journals

A basic structural check has been run on your CIF. These basic checks will be run on all CIFs submitted for publication in IUCr journals (*Acta Crystallographica*, *Journal of Applied Crystallography*, *Journal of Synchrotron Radiation*); however, if you intend to submit to *Acta Crystallographica Section C* or *E* or *IUCrData*, you should make sure that full publication checks are run on the final version of your CIF prior to submission.

Publication of your CIF in other journals

Please refer to the *Notes for Authors* of the relevant journal for any special instructions relating to CIF submission.

Validation response form

Please find below a validation response form (VRF) that can be filled in and pasted into your CIF.

```
# start Validation Reply Form
_vrf_PLAT183_hijazi3m
;
PROBLEM: Missing _cell_measurement_reflns_used value ....     Please Do !
RESPONSE: ...
;
_vrf_PLAT184_hijazi3m
;
PROBLEM: Missing _cell_measurement_theta_min value ......     Please Do !
RESPONSE: ...
;
_vrf_PLAT185_hijazi3m
;
PROBLEM: Missing _cell_measurement_theta_max value ......     Please Do !
RESPONSE: ...
;
_vrf_PLAT902_hijazi3m
;
PROBLEM: No (Interpretable) Reflections found in FCF ....     Please Check
RESPONSE: ...
```

;
_vrf_PLAT234_hijazi3m
;
PROBLEM: Large Hirshfeld Difference S1 -- C20 .. 0.29 Ang.
RESPONSE: ...
;
_vrf_PLAT306_hijazi3m
;
PROBLEM: Isolated Oxygen Atom (H-atoms Missing ?) .... O3W Check
RESPONSE: ...
;
_vrf_PLAT417_hijazi3m
;
PROBLEM: Short Inter D-H..H-D H2W1 .. H1W2 .. 2.04 Ang.
RESPONSE: ...
;
_vrf_PLAT430_hijazi3m
;
PROBLEM: Short Inter D...A Contact O3 .. O3W .. 2.82 Ang.
RESPONSE: ...
;
end Validation Reply Form

PLATON version of 06/05/2016; check.def file version of 05/05/2016

Datablock hijazi3m - ellipsoid plot

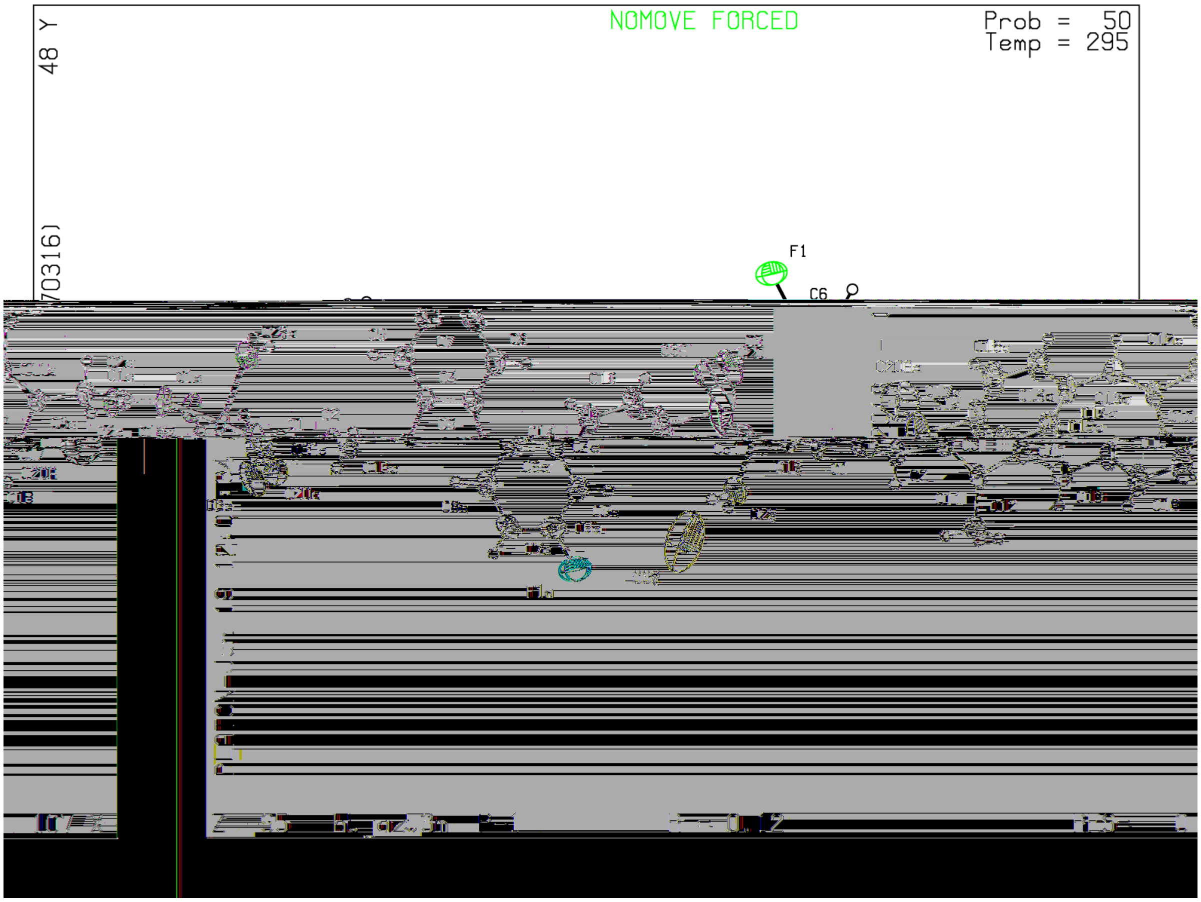

Supplement: Supplementary file 2 — Additional file 2: Table S2. Comparison between some of principle peaks in IR for K(sul) and 1 (cm-1). [file 13065_2017_268_MOESM2_ESM.pdf]
