# Supplementary material for: Synthesis, crystallographic, spectroscopic studies and biological activity of new cobalt(II) complexes with bioactive mixed sulindac and nitrogen-donor ligands
Source: Chem Cent J. 2017 May 10;11:40. doi: 10.1186/s13065-017-0268-2 (PMC5423883; doi:10.1186/s13065-017-0268-2)

checkCIF/PLATON report

Structure factors have been supplied for datablock(s) hijazi3m

THIS REPORT IS FOR GUIDANCE ONLY. IF USED AS PART OF A REVIEW PROCEDURE FOR PUBLICATION, IT SHOULD NOT REPLACE THE EXPERTISE OF AN EXPERIENCED CRYSTALLOGRAPHIC REFEREE.

No syntax errors found.   CIF dictionary   Interpreting this report

Datablock: hijazi3m

```
Bond precision:  C-C = 0.0185 A          Wavelength=0.71073

Cell:            a=20.930(3)     b=14.8360(19)       c=15.807(2)
                 alpha=90        beta=101.705(2)     gamma=90
Temperature:     295 K

                Calculated                  Reported
Volume          4806.3(11)                  4806.3(11)
Space group     P 21/c                      P2(1)/c
Hall group      -P 2ybc                     -P 2ybc
Moiety formula  C53 H38 Co F2 N2 O5 S2      C53 H38 Co F2 N2 O5 S2
Sum formula     C53 H38 Co F2 N2 O5 S2      C53 H38 Co F2 N2 O5 S2
Mr              943.90                      943.90
Dx,g cm-3       1.304                       1.304
Z               4                           4
Mu (mm-1)       0.500                       0.500
F000            1948.0                      1948.0
F000'           1951.22
h,k,lmax        26,18,20                    26,18,20
Nref            10501                       10468
Tmin,Tmax       0.767,0.975                 0.777,0.975
Tmin'           0.767

Correction method= # Reported T Limits: Tmin=0.777 Tmax=0.975
AbsCorr = MULTI-SCAN

Data completeness= 0.997          Theta(max)= 27.000

R(reflections)= 0.1941( 7302)     wR2(reflections)= 0.4718( 10468)

S = 1.575                  Npar= 603
```

The following ALERTS were generated. Each ALERT has the format
test-name_ALERT_alert-type_alert-level.
Click on the hyperlinks for more details of the test.

Alert level A

```
RFACR01_ALERT_3_A  The value of the weighted R factor is > 0.45
            Weighted R factor given   0.472
PLAT084_ALERT_3_A High wR2 Value (i.e. > 0.25) ...................       0.47 Report
PLAT215_ALERT_3_A Disordered C40       has ADP max/min Ratio .....        5.4 Note
PLAT234_ALERT_4_A Large Hirshfeld Difference S2     --  O6      ..       0.40 Ang.
PLAT234_ALERT_4_A Large Hirshfeld Difference S2     --  C40     ..       0.44 Ang.
PLAT234_ALERT_4_A Large Hirshfeld Difference C46    --  C47     ..       0.33 Ang.
PLAT241_ALERT_2_A High   'MainMol' Ueq as Compared to Neighbors of         S1 Check
PLAT902_ALERT_1_A No (Interpretable) Reflections found in FCF ....     Please Check
```

Alert level B

```
RFACG01_ALERT_3_B  The value of the R factor is > 0.15
            R factor given   0.194
PLAT082_ALERT_2_B High R1 Value ..................................       0.19 Report
PLAT213_ALERT_2_B Atom C47             has ADP max/min Ratio .....        4.7 prolat
PLAT234_ALERT_4_B Large Hirshfeld Difference C37    --  C38     ..       0.28 Ang.
PLAT241_ALERT_2_B High   'MainMol' Ueq as Compared to Neighbors of         S2 Check
PLAT241_ALERT_2_B High   'MainMol' Ueq as Compared to Neighbors of         O5 Check
PLAT242_ALERT_2_B Low    'MainMol' Ueq as Compared to Neighbors of        C17 Check
PLAT242_ALERT_2_B Low    'MainMol' Ueq as Compared to Neighbors of        C37 Check
PLAT341_ALERT_3_B Low Bond Precision on  C-C Bonds ...............     0.0185 Ang.
PLAT925_ALERT_1_B The Reported and Calculated Rho(max) Differ by .       2.15 eA-3
```

Alert level C

```
DIFMX01_ALERT_2_C  The maximum difference density is > 0.1*ZMAX*0.75
            _refine_diff_density_max given =      2.149
            Test value =      2.025
DIFMX02_ALERT_1_C  The maximum difference density is > 0.1*ZMAX*0.75
            The relevant atom site should be identified.
SHFSU01_ALERT_2_C  The absolute value of parameter shift to su ratio > 0.05
            Absolute value of the parameter shift to su ratio given   0.078
            Additional refinement cycles may be required.
PLAT080_ALERT_2_C Maximum Shift/Error ............................       0.08 Why ?
PLAT094_ALERT_2_C Ratio of Maximum / Minimum Residual Density ....       3.14 Report
PLAT097_ALERT_2_C Large Reported Max.  (Positive) Residual Density       2.15 eA-3
PLAT213_ALERT_2_C Atom S1              has ADP max/min Ratio .....        3.8 prolat
PLAT213_ALERT_2_C Atom C44             has ADP max/min Ratio .....        3.4 prolat
PLAT220_ALERT_2_C Non-Solvent Resd 1   C   Ueq(max)/Ueq(min) Range        4.3 Ratio
PLAT230_ALERT_2_C Hirshfeld Test Diff for    C45    --  C46     ..        7.0 s.u.
PLAT234_ALERT_4_C Large Hirshfeld Difference S1     --  C17     ..       0.21 Ang.
PLAT234_ALERT_4_C Large Hirshfeld Difference F2     --  C26     ..       0.25 Ang.
PLAT234_ALERT_4_C Large Hirshfeld Difference O5     --  C21     ..       0.22 Ang.
PLAT234_ALERT_4_C Large Hirshfeld Difference N1     --  C41     ..       0.17 Ang.
PLAT234_ALERT_4_C Large Hirshfeld Difference N2     --  C52     ..       0.18 Ang.
PLAT234_ALERT_4_C Large Hirshfeld Difference C7     --  C8      ..       0.16 Ang.
PLAT234_ALERT_4_C Large Hirshfeld Difference C22    --  C23     ..       0.18 Ang.
PLAT234_ALERT_4_C Large Hirshfeld Difference C23    --  C31     ..       0.18 Ang.
PLAT234_ALERT_4_C Large Hirshfeld Difference C24    --  C25     ..       0.23 Ang.
PLAT234_ALERT_4_C Large Hirshfeld Difference C25    --  C26     ..       0.25 Ang.
PLAT234_ALERT_4_C Large Hirshfeld Difference C28    --  C29     ..       0.16 Ang.
PLAT234_ALERT_4_C Large Hirshfeld Difference C30    --  C33     ..       0.18 Ang.
PLAT234_ALERT_4_C Large Hirshfeld Difference C34    --  C39     ..       0.18 Ang.
PLAT234_ALERT_4_C Large Hirshfeld Difference C36    --  C37     ..       0.24 Ang.
PLAT234_ALERT_4_C Large Hirshfeld Difference C38    --  C39     ..       0.22 Ang.
PLAT234_ALERT_4_C Large Hirshfeld Difference C43    --  C44     ..       0.20 Ang.
PLAT234_ALERT_4_C Large Hirshfeld Difference C44    --  C45     ..       0.18 Ang.
PLAT234_ALERT_4_C Large Hirshfeld Difference C51    --  C52     ..       0.23 Ang.
PLAT241_ALERT_2_C High   'MainMol' Ueq as Compared to Neighbors of         O1 Check
```

```
PLAT241_ALERT_2_C High   'MainMol' Ueq as Compared to Neighbors of         O2 Check
PLAT241_ALERT_2_C High   'MainMol' Ueq as Compared to Neighbors of         O4 Check
PLAT241_ALERT_2_C High   'MainMol' Ueq as Compared to Neighbors of        C22 Check
PLAT241_ALERT_2_C High   'MainMol' Ueq as Compared to Neighbors of        C25 Check
PLAT241_ALERT_2_C High   'MainMol' Ueq as Compared to Neighbors of        C33 Check
PLAT241_ALERT_2_C High   'MainMol' Ueq as Compared to Neighbors of        C35 Check
PLAT241_ALERT_2_C High   'MainMol' Ueq as Compared to Neighbors of        C38 Check
PLAT241_ALERT_2_C High   'MainMol' Ueq as Compared to Neighbors of        C44 Check
PLAT241_ALERT_2_C High   'MainMol' Ueq as Compared to Neighbors of        C46 Check
PLAT241_ALERT_2_C High   'MainMol' Ueq as Compared to Neighbors of        C47 Check
PLAT241_ALERT_2_C High   'MainMol' Ueq as Compared to Neighbors of        C50 Check
PLAT241_ALERT_2_C High   'MainMol' Ueq as Compared to Neighbors of        C51 Check
PLAT242_ALERT_2_C Low    'MainMol' Ueq as Compared to Neighbors of        Co1 Check
PLAT242_ALERT_2_C Low    'MainMol' Ueq as Compared to Neighbors of         C1 Check
PLAT242_ALERT_2_C Low    'MainMol' Ueq as Compared to Neighbors of        C21 Check
PLAT242_ALERT_2_C Low    'MainMol' Ueq as Compared to Neighbors of        C24 Check
PLAT242_ALERT_2_C Low    'MainMol' Ueq as Compared to Neighbors of        C26 Check
PLAT242_ALERT_2_C Low    'MainMol' Ueq as Compared to Neighbors of        C34 Check
PLAT242_ALERT_2_C Low    'MainMol' Ueq as Compared to Neighbors of        C42 Check
PLAT242_ALERT_2_C Low    'MainMol' Ueq as Compared to Neighbors of        C45 Check
PLAT242_ALERT_2_C Low    'MainMol' Ueq as Compared to Neighbors of        C48 Check
PLAT242_ALERT_2_C Low    'MainMol' Ueq as Compared to Neighbors of        C52 Check
PLAT362_ALERT_2_C Short   C(sp3)-C(sp2) Bond  C52    -   C54    ..       1.41 Ang.
PLAT369_ALERT_2_C Long    C(sp2)-C(sp2) Bond  C47    -   C48    ..       1.56 Ang.
PLAT601_ALERT_2_C Structure Contains Solvent Accessible VOIDS of .         46 Ang3
```

Alert level G

```
PLAT005_ALERT_5_G No Embedded Refinement Details found  in the CIF     Please Do !
PLAT066_ALERT_1_G Predicted and Reported Tmin&Tmax Range Identical          ? Check
PLAT072_ALERT_2_G SHELXL First  Parameter in WGHT  Unusually Large       0.20 Report
PLAT093_ALERT_1_G No s.u.'s on H-positions, Refinement Reported as      mixed Check
PLAT230_ALERT_2_G Hirshfeld Test Diff for    S1     --  O3      ..       14.0 s.u.
PLAT232_ALERT_2_G Hirshfeld Test Diff (M-X)  Co1    --  O5      ..        5.7 s.u.
PLAT300_ALERT_4_G Atom Site Occupancy of *O3     is Constrained at        0.5 Check
PLAT300_ALERT_4_G Atom Site Occupancy of *O6     is Constrained at        0.5 Check
PLAT300_ALERT_4_G Atom Site Occupancy of *C20    is Constrained at        0.5 Check
PLAT300_ALERT_4_G Atom Site Occupancy of *C40    is Constrained at        0.5 Check
PLAT301_ALERT_3_G Main Residue  Disorder ............ Percentage =          3 Note
PLAT333_ALERT_2_G Check Large Av C6-Ring C-C Dist. C41    -C49           1.44 Ang.
PLAT335_ALERT_2_G Check Large C6 Ring    C-C Range C24    -C29           0.18 Ang.
PLAT335_ALERT_2_G Check Large C6 Ring    C-C Range C41    -C49           0.32 Ang.
PLAT899_ALERT_4_G SHELXL97   is Deprecated and Succeeded by SHELXL       2014 Note
```

---

  8 **ALERT level A** = Most likely a serious problem - resolve or explain
 10 **ALERT level B** = A potentially serious problem, consider carefully
 54 **ALERT level C** = Check. Ensure it is not caused by an omission or oversight
 15 **ALERT level G** = General information/check it is not something unexpected

```
  5 ALERT type 1 CIF construction/syntax error, inconsistent or missing data
 48 ALERT type 2 Indicator that the structure model may be wrong or deficient
  6 ALERT type 3 Indicator that the structure quality may be low
 27 ALERT type 4 Improvement, methodology, query or suggestion
  1 ALERT type 5 Informative message, check
```

It is advisable to attempt to resolve as many as possible of the alerts in all categories. Often the minor alerts point to easily fixed oversights, errors and omissions in your CIF or refinement strategy, so attention to these fine details can be worthwhile. In order to resolve some of the more serious problems it may be necessary to carry out additional measurements or structure refinements. However, the purpose of your study may justify the reported deviations and the more serious of these should normally be commented upon in the discussion or experimental section of a paper or in the "special_details" fields of the CIF. checkCIF was carefully designed to identify outliers and unusual parameters, but every test has its limitations and alerts that are not important in a particular case may appear. Conversely, the absence of alerts does not guarantee there are no aspects of the results needing attention. It is up to the individual to critically assess their own results and, if necessary, seek expert advice.

Publication of your CIF in IUCr journals

A basic structural check has been run on your CIF. These basic checks will be run on all CIFs submitted for publication in IUCr journals (*Acta Crystallographica*, *Journal of Applied Crystallography*, *Journal of Synchrotron Radiation*); however, if you intend to submit to *Acta Crystallographica Section C* or *E* or *IUCrData*, you should make sure that full publication checks are run on the final version of your CIF prior to submission.

Publication of your CIF in other journals

Please refer to the *Notes for Authors* of the relevant journal for any special instructions relating to CIF submission.

Validation response form

Please find below a validation response form (VRF) that can be filled in and pasted into your CIF.

```
# start Validation Reply Form
_vrf_RFACR01_hijazi3m
;
PROBLEM: The value of the weighted R factor is > 0.45
RESPONSE: ...
;
_vrf_DIFMX01_hijazi3m
;
PROBLEM: The maximum difference density is > 0.1*ZMAX*0.75
RESPONSE: ...
;
_vrf_DIFMX02_hijazi3m
;
PROBLEM: The maximum difference density is > 0.1*ZMAX*0.75
RESPONSE: ...
;
_vrf_SHFSU01_hijazi3m
;
PROBLEM: The absolute value of parameter shift to su ratio > 0.05
RESPONSE: ...
;
_vrf_PLAT084_hijazi3m
;
PROBLEM: High wR2 Value (i.e. > 0.25) ...................       0.47 Report
RESPONSE: ...
;
_vrf_PLAT215_hijazi3m
```

```
;
PROBLEM: Disordered C40       has ADP max/min Ratio .....        5.4 Note
RESPONSE: ...
;
_vrf_PLAT234_hijazi3m
;
PROBLEM: Large Hirshfeld Difference S2     --  O6      ..       0.40 Ang.
RESPONSE: ...
;
_vrf_PLAT241_hijazi3m
;
PROBLEM: High   'MainMol' Ueq as Compared to Neighbors of         S1 Check
RESPONSE: ...
;
_vrf_PLAT902_hijazi3m
;
PROBLEM: No (Interpretable) Reflections found in FCF ....     Please Check
RESPONSE: ...
;
_vrf_PLAT080_hijazi3m
;
PROBLEM: Maximum Shift/Error ...........................       0.08 Why ?
RESPONSE: ...
;
_vrf_PLAT094_hijazi3m
;
PROBLEM: Ratio of Maximum / Minimum Residual Density ....       3.14 Report
RESPONSE: ...
;
_vrf_PLAT097_hijazi3m
;
PROBLEM: Large Reported Max.  (Positive) Residual Density       2.15 eA-3
RESPONSE: ...
;
_vrf_PLAT213_hijazi3m
;
PROBLEM: Atom S1              has ADP max/min Ratio .....        3.8 prolat
RESPONSE: ...
;
_vrf_PLAT220_hijazi3m
;
PROBLEM: Non-Solvent Resd 1   C   Ueq(max)/Ueq(min) Range        4.3 Ratio
RESPONSE: ...
;
_vrf_PLAT230_hijazi3m
;
PROBLEM: Hirshfeld Test Diff for    C45    --  C46     ..        7.0 s.u.
RESPONSE: ...
;
_vrf_PLAT242_hijazi3m
;
PROBLEM: Low    'MainMol' Ueq as Compared to Neighbors of        Co1 Check
RESPONSE: ...
;
_vrf_PLAT362_hijazi3m
;
PROBLEM: Short   C(sp3)-C(sp2) Bond  C52    -   C54    ..       1.41 Ang.
RESPONSE: ...
;
_vrf_PLAT369_hijazi3m
;
PROBLEM: Long    C(sp2)-C(sp2) Bond  C47    -   C48    ..       1.56 Ang.
```

```
RESPONSE: ...
;
_vrf_PLAT601_hijazi3m
;
PROBLEM: Structure Contains Solvent Accessible VOIDS of .         46 Ang3
RESPONSE: ...
;
# end Validation Reply Form
```

PLATON version of 11/08/2016; check.def file version of 04/08/2016

Datablock hijazi3m - ellipsoid plot

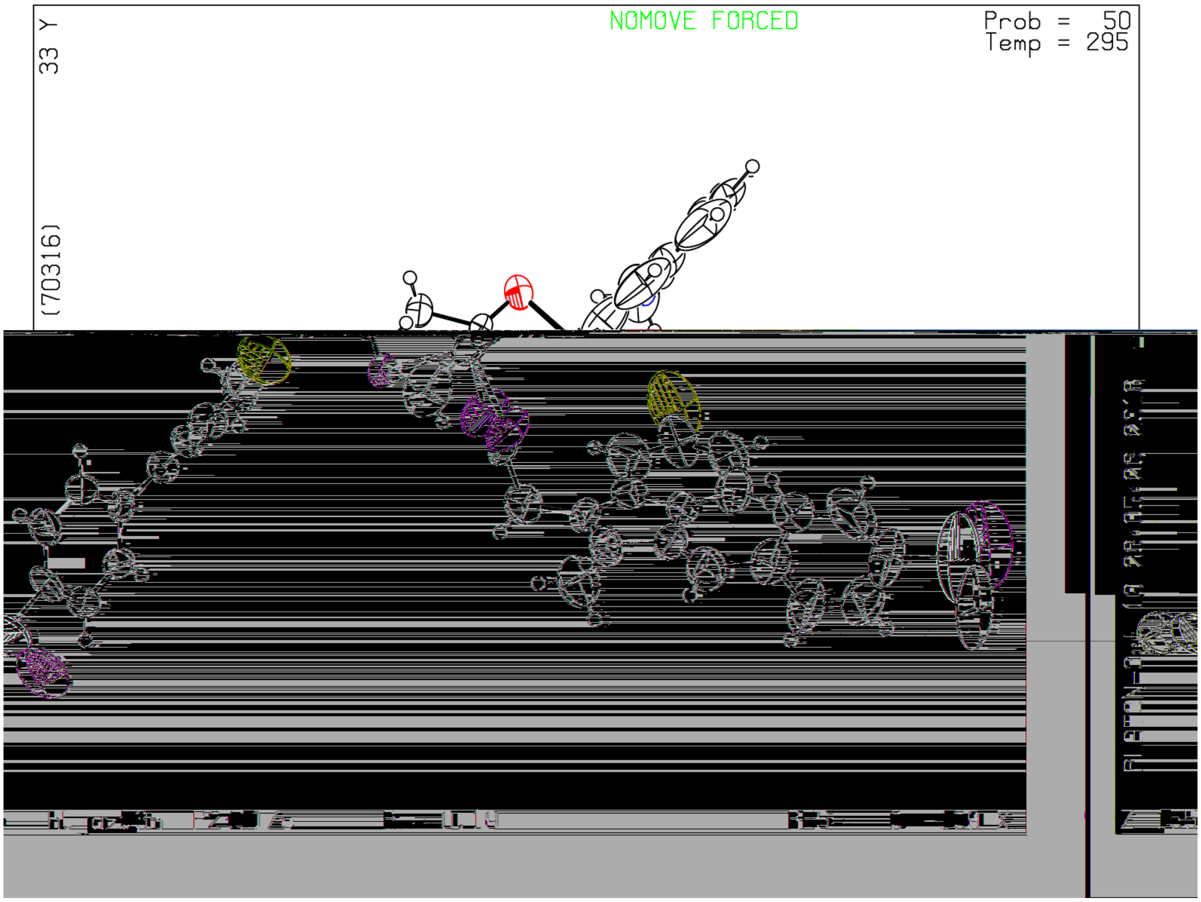

Supplement: Supplementary file 4 — Additional file 4: Table S4. UV-visible spectral data for compounds (1–4). [file 13065_2017_268_MOESM4_ESM.pdf]
