# Supplementary material for: Synthesis, crystallographic, spectroscopic studies and biological activity of new cobalt(II) complexes with bioactive mixed sulindac and nitrogen-donor ligands
Source: Chem Cent J. 2017 May 10;11:40. doi: 10.1186/s13065-017-0268-2 (PMC5423883; doi:10.1186/s13065-017-0268-2)
Supplement: Supplementary file 5 — Additional file 5: CCDC 1450310 and CCDC 1450311 contain the supplementary crystallographic data for complexes 1 and 4. These data can be obtained free of charge via http://www.ccdc.cam.ac.uk/conts/retrieving.html, or from the Cambridge Crystallographic Data Centre, 12 Union Road, Cambridge CB2 1EZ, UK; fax: (+44) 1223-336-033; or e-mail: deposit@ccdc.cam.ac.uk. Supplementary data associated with this article can be found, in the online version. [file 13065_2017_268_MOESM5_ESM.docx]

**Appendix A. Supplementary data**

**Table S1:** Physical properties and yield of Cobalt(II) sulindac compounds.

| Compounds | %Yield | m.p (°C) | Solubility |
| --- | --- | --- | --- |
| [Co(sul)_2_.4H_2_O] (**1**) | 85 | 201 | MeOH, DMF, DMSO |
| [Co(sul)_2_ (2-ampy)_2_] (**2**) | 56 | 180^d^ | MeOH, DMF, DMSO |
| [Co(sul)_2_(1,10-phen)] (**3**) | 22 | 140 | MeOH, CHCl_3_, DCM, DMF, DMSO |
| [Co(sul)_2_(2,9-dimephen)] (**4**) | 34 | 150^d^ | MeOH, CHCl_3_, DCM, DMF, DMSO |

d: decomposition

**Table S2:** Comparison between some of principle peaks in IR for K(sul) and **1** (cm^-1^).

| Assignments | K(sul) | Complex **1** |
| --- | --- | --- |
| υ (C-H)_ar_ | 3066 | 3050 |
| υ (C-H)_aliph_ | 2970, 2880 | 2911, 2850 |
| υ_as_(COO^-^) | 1576 | 1600 |
| υ_s_(COO^-^) | 1398 | 1416 |
| υ (ring)+δ (C-H) | 1476, 1367 | 1465, 1369 |
| Δυ (COO^-^) | 178 | 184 |

**Table S3:** Summary of principle peaks in IR for complexes **2, 3** and **4** (cm^-1^)

| Assignments | Complex **2** | Complex **3** | Complex **4** |
| --- | --- | --- | --- |
| υ_as_(N-H) | 3374 | - | - |
| υ_s_(N-H) | 3268 | - | - |
| υ(C-H)_ar_ | 3015 | 3059 | 3040 |
| υ(C-H)_aliph_ | 2914, 2860 | 2911, 2852 | 2912, 2845 |
| υ(ring) | 1599 | 1600 | 1599 |
| υ(ring) + δ(C-H) | 1464, 1424 | 1464, 1424 | 1465, 1359 |
| υ_s_ (COO^-^) | 1380 | 1380 | 1441 |
| υ(ring) | 1267, 1086 | 1267, 1086 | 1194, 1086 |
| γ(C-H) | 891 | 846 | 855 |
| δ(COO^-^) | 727 | 727 | 761, 728 |
| Δ(COO^-^) | 219 | 220 | 158 |

**Table S4:** UV-visible spectral data for compounds (**1-4**).

| Compounds | λ_max_ (nm) | *ε* (Lmol^-1^cm^-1^) |
| --- | --- | --- |
| [Co(sul)_2_.4H_2_O] (**1**) | 211  258  264  252  282  328 | 3283  872  850  828  771  514 |
| [Co(sul)_2_(2-ampy)_2_] (**2**) | 207  286  329  655 | 1828  450  348  12.7 |
| [Co(sul)_2_(1,10-phen)] (**3**) | 208  226  271  328  431  488 | 2152  700  535  224  16.3  13.2 |
| [Co(sul)_2_(2,9-dimephen)] (**4**) | 207  229  274  328  432 | 2263  933  621  261  13.3 |
